# Poly[(μ_4_-biphenyl-2,4′-dicarboxyl­ato-κ^5^O^2^:O^2′^:O^4^:O^4^,O^4′^)zinc]

**DOI:** 10.1107/S1600536812038901

**Published:** 2012-09-19

**Authors:** Yu-Guang Tian

**Affiliations:** a College of Pharmaceutical Engineering, Guangdong Food and Drug Vocational College, Guangzhou 510520, People’s Republic of China

## Abstract

The crystal structure of the polymeric title complex, [Zn(C_14_H_8_O_4_)]_*n*_, is composed of layers parallel to (110) formed by linking of Zn–carboxyl­ate chains with biphenyl units of the biphenyl-2,4′-dicarboxyl­ate (bpdc) ligands. The Zn^II^ atom is five-coordinated in a distorted square-pyramidal geometry by five O atoms from four bpdc ligands. The dihedral angle between the benzene rings is 52.32 (12)°.

## Related literature

For related structures, see: Guo *et al.* (2010 ▶); Jia *et al.* (2011 ▶); Zhang *et al.* (2011 ▶).
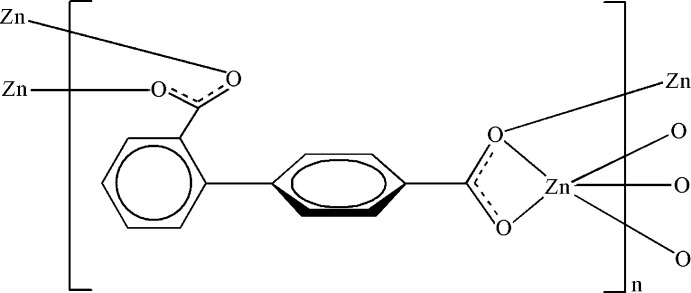


## Experimental

### 

#### Crystal data

[Zn(C_14_H_8_O_4_)]
*M* *_r_* = 305.59
Orthorhombic, 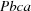

*a* = 12.702 (8) Å
*b* = 7.178 (4) Å
*c* = 25.368 (15) Å
*V* = 2313 (2) Å^3^
*Z* = 8
Mo *K*α radiation
μ = 2.13 mm^−1^
*T* = 296 K
0.25 × 0.20 × 0.18 mm

#### Data collection

Bruker APEXII CCD diffractometer
Absorption correction: multi-scan (*SADABS*; Sheldrick, 1996 ▶) *T*_min_ = 0.606, *T*_max_ = 0.682
10978 measured reflections
2080 independent reflections
1453 reflections with *I* > 2σ(*I*)
*R*_int_ = 0.060

#### Refinement

*R*[*F* ^2^ > 2σ(*F* ^2^)] = 0.036
*wR*(*F* ^2^) = 0.086
*S* = 1.06
2080 reflections
172 parameters
1 restraint
H-atom parameters constrained
Δρ_max_ = 0.43 e Å^−3^
Δρ_min_ = −0.44 e Å^−3^

### 

Data collection: *APEX2* (Bruker, 2007 ▶); cell refinement: *SAINT* (Bruker, 2007 ▶); data reduction: *SAINT*; program(s) used to solve structure: *SHELXS97* (Sheldrick, 2008 ▶); program(s) used to refine structure: *SHELXL97* (Sheldrick, 2008 ▶); molecular graphics: *DIAMOND* (Brandenburg, 1999 ▶); software used to prepare material for publication: *SHELXTL* (Sheldrick, 2008 ▶).

## Supplementary Material

Crystal structure: contains datablock(s) I, global. DOI: 10.1107/S1600536812038901/hy2581sup1.cif

Structure factors: contains datablock(s) I. DOI: 10.1107/S1600536812038901/hy2581Isup2.hkl

Additional supplementary materials:  crystallographic information; 3D view; checkCIF report

## Figures and Tables

**Table 1:** Selected bond lengths (Å)

Zn1—O1^i^	1.971 (3)
Zn1—O2^ii^	1.937 (3)
Zn1—O3	2.130 (3)
Zn1—O3^iii^	2.003 (3)
Zn1—O4	2.204 (3)

Symmetry codes: (i) 
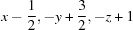
; (ii) 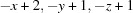; (iii) 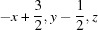.

**Fig. 1.**
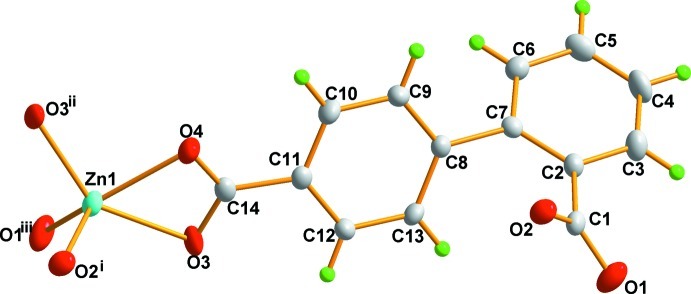
The asymmetric unit of the title compound. Displacement ellipsoids are drawn at the 50% probability level. [Symmetry codes: (i) 2-x, 1-y, 1-z; (ii) 3/2-x, -1/2+y, z; (iii) -1/2+x, 3/2-y, 1-z.]

**Fig. 2.**
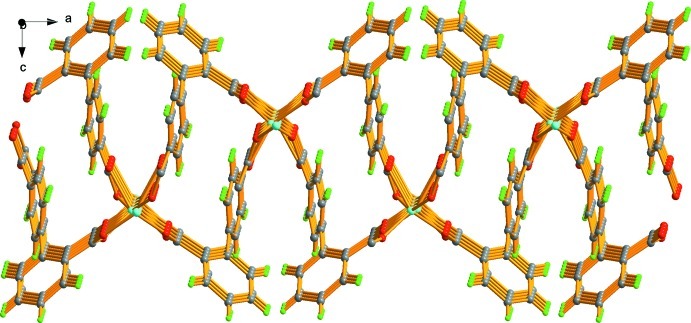
A view of the layer structure in the title compound.

## supplementary crystallographic information

### Comment

In recent years, the design and synthesis of metal-oganic frameworks (MOFs) are of great interest in the view of their fascinating structural diversity and significance of discovering new materials in the field of catalysis, gas storage, fluorescence, magnetism and so on. However, how to choose the metal centers and multidentate ligands is still a challedge. Biphenyl-2,4'-dicarboxylic acid (H_2_bpdc) can be utilized as a multifunctional bridging ligand because it can bridge metal centers to form chains, layers or three-dimensional networks. The rotation of the C—C single bond between the two phenyl rings gives rise to a skew coordination orientation of the carboxylate groups, which is favorable for the formation of various new complexes with intriguing architectures and topologies (Guo *et al.*, 2010; Jia *et al.*, 2011; Zhang *et al.*, 2011). Recently, we synthesized the title coordination polymer under hydrothermal conditions.

In the title compound (Fig. 1), the bpdc ligand is fully deprotonated. The Zn^II^ atom is five-coordinated by five O atoms from four different bpdc ligands in a distorted square-pyramidal geometry, with Zn—O distances and O—Zn—O angles ranging from 1.937 (2) to 2.204 (2) Å (Table 1) and 92.02 (11) to 108.20 (13)°, respectively. Adjacent Zn^II^ atoms are bridged by a bidentate carboxylate group and a tridentate carboxylate group, which come from two different bpdc ligands, forming a Zn-carboxylate chain. These chains are further linked by the bpdc ligands into a layer structure (Fig. 2).

### Experimental

A mixture of ZnCl_2_ (0.068 g, 0.5 mmol), biphenyl-2,4'-dicarboxylic acid (0.121 g, 0.5 mmol) and water (8 ml) in the presence of CH_3_COOH (2 ml) was stirred vigorously for 30 min and then sealed in a 20 ml Teflon-lined stainless-steel autoclave. The autoclave was heated and maintained at 393 K for 3 days, and then cooled to room temperature at 5 K h^-1^ to yield colorless block crystals.

### Refinement

H atoms were positioned geometrically and refined as riding atoms, with C—H = 0.93 Å and with *U*_iso_(H) = 1.2*U*_eq_(C).

### Crystal data

**Table:**

[Zn(C_14_H_8_O_4_)]	*F*(000) = 1232.0
*M**_r_* = 305.59	*D*_x_ = 1.755 Mg m^−^^3^
Orthorhombic, *P**b**c**a*	Mo *K*α radiation, λ = 0.71073 Å
Hall symbol: -P 2ac 2ab	Cell parameters from 1609 reflections
*a* = 12.702 (8) Å	θ = 2.3–21.7°
*b* = 7.178 (4) Å	µ = 2.13 mm^−^^1^
*c* = 25.368 (15) Å	*T* = 296 K
*V* = 2313 (2) Å^3^	Block, colourless
*Z* = 8	0.25 × 0.20 × 0.18 mm

### Data collection

**Table:**

Bruker APEXII CCD diffractometer	2080 independent reflections
Radiation source: fine-focus sealed tube	1453 reflections with *I* > 2σ(*I*)
Graphite monochromator	*R*_int_ = 0.060
φ and ω scans	θ_max_ = 25.2°, θ_min_ = 1.6°
Absorption correction: multi-scan (*SADABS*; Sheldrick, 1996)	*h* = −15→15
*T*_min_ = 0.606, *T*_max_ = 0.682	*k* = −8→8
10978 measured reflections	*l* = −24→30

### Refinement

**Table:**

Refinement on *F*^2^	Primary atom site location: structure-invariant direct methods
Least-squares matrix: full	Secondary atom site location: difference Fourier map
*R*[*F*^2^ > 2σ(*F*^2^)] = 0.036	Hydrogen site location: inferred from neighbouring sites
*wR*(*F*^2^) = 0.086	H-atom parameters constrained
*S* = 1.06	*w* = 1/[σ^2^(*F*_o_^2^) + (0.031*P*)^2^ + 1.2131*P*] where *P* = (*F*_o_^2^ + 2*F*_c_^2^)/3
2080 reflections	(Δ/σ)_max_ = 0.001
172 parameters	Δρ_max_ = 0.43 e Å^−^^3^
1 restraint	Δρ_min_ = −0.44 e Å^−^^3^

### Special details

**Table:**

Geometry. All e.s.d.'s (except the e.s.d. in the dihedral angle between two l.s. planes) are estimated using the full covariance matrix. The cell e.s.d.'s are taken into account individually in the estimation of e.s.d.'s in distances, angles and torsion angles; correlations between e.s.d.'s in cell parameters are only used when they are defined by crystal symmetry. An approximate (isotropic) treatment of cell e.s.d.'s is used for estimating e.s.d.'s involving l.s. planes.
Refinement. Refinement of *F*^2^ against ALL reflections. The weighted *R*-factor *wR* and goodness of fit *S* are based on *F*^2^, conventional *R*-factors *R* are based on *F*, with *F* set to zero for negative *F*^2^. The threshold expression of *F*^2^ > σ(*F*^2^) is used only for calculating *R*-factors(gt) *etc*. and is not relevant to the choice of reflections for refinement. *R*-factors based on *F*^2^ are statistically about twice as large as those based on *F*, and *R*- factors based on ALL data will be even larger.

### Fractional atomic coordinates and isotropic or equivalent isotropic displacement parameters (Å^2^)

**Table:**

	*x*	*y*	*z*	*U*_iso_*/*U*_eq_	
Zn1	0.75928 (3)	0.20620 (6)	0.422620 (17)	0.03431 (17)	
C1	1.0994 (3)	1.0497 (6)	0.63381 (14)	0.0321 (9)	
C2	1.0009 (3)	1.0593 (5)	0.66637 (13)	0.0296 (9)	
C3	0.9958 (3)	1.1891 (6)	0.70684 (16)	0.0443 (11)	
H3	1.0484	1.2787	0.7097	0.053*	
C4	0.9143 (3)	1.1879 (6)	0.74286 (16)	0.0499 (12)	
H4	0.9134	1.2734	0.7704	0.060*	
C5	0.8346 (3)	1.0598 (6)	0.73783 (15)	0.0472 (11)	
H5	0.7793	1.0581	0.7619	0.057*	
C6	0.8373 (3)	0.9341 (5)	0.69682 (15)	0.0371 (10)	
H6	0.7823	0.8497	0.6932	0.044*	
C7	0.9202 (3)	0.9289 (5)	0.66038 (13)	0.0289 (9)	
C8	0.9135 (3)	0.7914 (5)	0.61639 (14)	0.0296 (8)	
C9	0.8928 (3)	0.6051 (6)	0.62683 (15)	0.0381 (10)	
H9	0.8907	0.5633	0.6615	0.046*	
C10	0.8751 (3)	0.4808 (6)	0.58591 (15)	0.0412 (10)	
H10	0.8618	0.3561	0.5934	0.049*	
C11	0.8772 (3)	0.5406 (5)	0.53420 (14)	0.0319 (9)	
C12	0.9020 (3)	0.7254 (5)	0.52361 (14)	0.0330 (9)	
H12	0.9066	0.7658	0.4889	0.040*	
C13	0.9201 (3)	0.8498 (5)	0.56432 (14)	0.0333 (9)	
H13	0.9367	0.9731	0.5567	0.040*	
C14	0.8443 (3)	0.4097 (6)	0.49200 (15)	0.0337 (9)	
O1	1.1384 (2)	1.2024 (4)	0.61869 (12)	0.0545 (8)	
O2	1.13685 (19)	0.8913 (4)	0.62598 (10)	0.0400 (7)	
O3	0.8129 (2)	0.4737 (3)	0.44709 (10)	0.0398 (6)	
O4	0.8418 (2)	0.2388 (4)	0.49848 (11)	0.0462 (7)	

### Atomic displacement parameters (Å^2^)

**Table:**

	*U*^11^	*U*^22^	*U*^33^	*U*^12^	*U*^13^	*U*^23^
Zn1	0.0357 (3)	0.0219 (3)	0.0453 (3)	−0.0005 (2)	0.0018 (2)	−0.0084 (2)
C1	0.031 (2)	0.031 (2)	0.035 (2)	−0.0027 (19)	−0.0082 (17)	−0.0021 (18)
C2	0.030 (2)	0.027 (2)	0.032 (2)	0.0031 (16)	−0.0043 (16)	−0.0039 (18)
C3	0.041 (2)	0.040 (3)	0.051 (3)	0.003 (2)	−0.008 (2)	−0.013 (2)
C4	0.055 (3)	0.054 (3)	0.041 (3)	0.012 (2)	−0.005 (2)	−0.021 (2)
C5	0.048 (3)	0.055 (3)	0.039 (3)	0.017 (2)	0.005 (2)	−0.003 (2)
C6	0.036 (2)	0.034 (3)	0.041 (2)	−0.0001 (18)	−0.0007 (18)	0.0013 (19)
C7	0.032 (2)	0.028 (2)	0.027 (2)	0.0044 (16)	−0.0030 (16)	0.0012 (16)
C8	0.0271 (19)	0.031 (2)	0.031 (2)	−0.0021 (17)	−0.0023 (16)	−0.0023 (18)
C9	0.047 (3)	0.037 (2)	0.031 (2)	−0.009 (2)	−0.0028 (18)	0.0033 (19)
C10	0.051 (3)	0.027 (2)	0.046 (3)	−0.0061 (19)	−0.007 (2)	0.001 (2)
C11	0.026 (2)	0.034 (2)	0.035 (2)	−0.0012 (17)	−0.0001 (16)	−0.0038 (19)
C12	0.037 (2)	0.035 (2)	0.028 (2)	−0.0041 (18)	−0.0029 (17)	0.0002 (18)
C13	0.037 (2)	0.024 (2)	0.040 (2)	−0.0066 (17)	−0.0041 (17)	0.0019 (18)
C14	0.026 (2)	0.040 (3)	0.034 (2)	0.0002 (18)	0.0010 (17)	−0.007 (2)
O1	0.0431 (17)	0.0318 (18)	0.088 (2)	−0.0026 (14)	0.0184 (15)	−0.0057 (16)
O2	0.0440 (16)	0.0259 (15)	0.0502 (17)	0.0058 (13)	0.0097 (13)	−0.0021 (13)
O3	0.0527 (17)	0.0279 (12)	0.0388 (16)	0.0081 (12)	−0.0096 (14)	−0.0153 (13)
O4	0.0600 (19)	0.0300 (17)	0.0486 (18)	−0.0129 (14)	−0.0018 (14)	−0.0046 (13)

### Geometric parameters (Å, º)

**Table:**

Zn1—O1^i^	1.971 (3)	C6—C7	1.401 (5)
Zn1—O2^ii^	1.937 (3)	C6—H6	0.9300
Zn1—O3	2.130 (3)	C7—C8	1.493 (5)
Zn1—O3^iii^	2.003 (3)	C8—C13	1.388 (5)
Zn1—O4	2.204 (3)	C8—C9	1.389 (5)
C1—O2	1.249 (4)	C9—C10	1.387 (5)
C1—O1	1.263 (4)	C9—H9	0.9300
C1—C2	1.501 (5)	C10—C11	1.381 (5)
C2—C3	1.388 (5)	C10—H10	0.9300
C2—C7	1.396 (5)	C11—C12	1.390 (5)
C3—C4	1.380 (6)	C11—C14	1.484 (5)
C3—H3	0.9300	C12—C13	1.384 (5)
C4—C5	1.374 (6)	C12—H12	0.9300
C4—H4	0.9300	C13—H13	0.9300
C5—C6	1.378 (5)	C14—O4	1.238 (5)
C5—H5	0.9300	C14—O3	1.292 (4)
			
O2^ii^—Zn1—O1^i^	108.20 (13)	C2—C7—C8	124.5 (3)
O2^ii^—Zn1—O3^iii^	102.00 (11)	C6—C7—C8	117.9 (3)
O1^i^—Zn1—O3^iii^	94.92 (12)	C13—C8—C9	118.9 (3)
O2^ii^—Zn1—O3	107.08 (11)	C13—C8—C7	120.6 (3)
O1^i^—Zn1—O3	95.97 (11)	C9—C8—C7	120.3 (3)
O3^iii^—Zn1—O3	143.83 (14)	C10—C9—C8	120.5 (4)
O2^ii^—Zn1—O4	105.69 (11)	C10—C9—H9	119.8
O1^i^—Zn1—O4	143.07 (12)	C8—C9—H9	119.8
O3^iii^—Zn1—O4	92.02 (11)	C11—C10—C9	120.5 (4)
O3—Zn1—O4	59.85 (10)	C11—C10—H10	119.7
O2—C1—O1	126.3 (4)	C9—C10—H10	119.7
O2—C1—C2	116.6 (3)	C10—C11—C12	119.0 (3)
O1—C1—C2	117.0 (3)	C10—C11—C14	118.9 (4)
C3—C2—C7	119.7 (4)	C12—C11—C14	121.9 (3)
C3—C2—C1	118.5 (3)	C13—C12—C11	120.6 (3)
C7—C2—C1	121.4 (3)	C13—C12—H12	119.7
C4—C3—C2	121.4 (4)	C11—C12—H12	119.7
C4—C3—H3	119.3	C12—C13—C8	120.3 (4)
C2—C3—H3	119.3	C12—C13—H13	119.8
C5—C4—C3	119.7 (4)	C8—C13—H13	119.8
C5—C4—H4	120.2	O4—C14—O3	117.5 (3)
C3—C4—H4	120.2	O4—C14—C11	122.6 (4)
C4—C5—C6	119.3 (4)	O3—C14—C11	119.9 (4)
C4—C5—H5	120.3	C1—O1—Zn1^iv^	139.1 (3)
C6—C5—H5	120.3	C1—O2—Zn1^ii^	133.6 (3)
C5—C6—C7	122.3 (4)	C14—O3—Zn1^v^	135.3 (2)
C5—C6—H6	118.9	C14—O3—Zn1	92.0 (2)
C7—C6—H6	118.9	Zn1^v^—O3—Zn1	120.98 (13)
C2—C7—C6	117.5 (3)	C14—O4—Zn1	90.1 (2)

Symmetry codes: (i) *x*−1/2, −*y*+3/2, −*z*+1; (ii) −*x*+2, −*y*+1, −*z*+1; (iii) −*x*+3/2, *y*−1/2, *z*; (iv) *x*+1/2, −*y*+3/2, −*z*+1; (v) −*x*+3/2, *y*+1/2, *z*.
